# Development of bioluminescent chick chorioallantoic membrane (CAM) models for primary pancreatic cancer cells: a platform for drug testing

**DOI:** 10.1038/srep44686

**Published:** 2017-03-17

**Authors:** Maria Rovithi, Amir Avan, Niccola Funel, Leticia G. Leon, Valentina E. Gomez, Thomas Wurdinger, Arjan W. Griffioen, Henk M. W. Verheul, Elisa Giovannetti

**Affiliations:** 1 Department of Medical Oncology, Cancer Center Amsterdam, VU University Medical Center, Amsterdam, The Netherlands; 2 Department of Internal Medicine, Agios Nikolaos General Hospital, Agios Nikolaos, Crete, Greece; 3 Molecular Medicine Group, Department of Modern Sciences and Technologies; School of Medicine, Mashhad University of Medical Sciences, Mashhad, Iran; 4 Cancer Pharmacology Lab, AIRC Start-Up Unit, University of Pisa, Pisa, Italy; 5 Department of Neurosurgery, Cancer Center Amsterdam, VU University Medical Center, Amsterdam, The Netherlands; 6 Molecular Neurogenetics Unit, Department of Neurology, Massachusetts General Hospital and Neuroscience Program, Harvard Medical School, Boston, Massachusetts, US

## Abstract

The aim of the present study was to develop chick-embryo chorioallantoic membrane (CAM) bioluminescent tumor models employing low passage cell cultures obtained from primary pancreatic ductal adenocarcinoma (PDAC) cells. Primary PDAC cells transduced with lentivirus expressing Firefly-luciferase (Fluc) were established and inoculated onto the CAM membrane, with >80% engraftment. Fluc signal reliably correlated with tumor growth. Tumor features were evaluated by immunohistochemistry and genetic analyses, including analysis of mutations and mRNA expression of PDAC pivotal genes, as well as microRNA (miRNA) profiling. These studies showed that CAM tumors had histopathological and genetic characteristic comparable to the original tumors. We subsequently tested the modulation of key miRNAs and the activity of gemcitabine and crizotinib on CAM tumors, showing that combination treatment resulted in 63% inhibition of tumor growth as compared to control (*p* < 0.01). These results were associated with reduced expression of miR-21 and increased expression of miR-155. Our study provides the first evidence that transduced primary PDAC cells can form tumors on the CAM, retaining several histopathological and (epi)genetic characteristics of original tumors. Moreover, our results support the use of these models for drug testing, providing insights on molecular mechanisms underlying antitumor activity of new drugs/combinations.

With less than 7% of patients alive five years after diagnosis, pancreatic ductal adenocarcinoma (PDAC) exhibits one of the poorest prognoses of all solid tumors. Despite extensive clinical efforts, the outcome of this malignancy has not improved in the last decade, and PDAC is expected to become the second deadliest cancer, after lung cancer, by 2030^1^,^2^.

Gaining more insight into the mechanisms that delineate tumor progression in PDAC could ultimately provide more successful therapeutic approaches. To this end, genetically engineered mouse models (GEMMs) have provided a powerful tool, developing tumors that recapitulate both the underlying biology and the dense desmoplastic reaction of PDACs. This stromal reaction has been considered for years as one of the mediators of resistance to chemotherapy^3^. However, experimental and clinical evidence demonstrated that anti-stromal approaches may favour PDAC aggressiveness, reinforcing the need to critically revisit the complexity of cancer-stroma interactions for translational and pharmacological implications^4^. Recent studies suggested that early passages of primary PDAC cells and “avatar” mice can mimic the genetic diversity that characterizes the human disease and might be better predictors of drug activity, including the standard treatment with gemcitabine^5^,^6^.

Despite several studies used such *in vivo* models in order to promote drug development and selection, their costs and complexity impaired the translation of these results in the clinical setting. Novel, cost-effective models that similarly mimic tumor biology and provide faster information on the activity of anticancer therapies could therefore make an important contribution to the advancement of personalized medicine.

The chicken embryo chorioallantoic membrane (CAM) assay has been widely used to study neovascularization^7^. However, CAM provides a uniquely supportive environment to study not only angiogenesis, but also tumorigenesis. More recently, the CAM assay has indeed been modified to work as an *in vivo* xenograft model system for various cancers, including PDAC^8^,^9^.

A major challenge for the further development of the CAM model consists in the validation of an appropriate method to evaluate tumor growth. Previous studies assessed tumor dimension through size measurements and weight as well as total tumor cell counts^10^,^11^,^12^. These methods could potentially be complemented with bioluminescence (BLI), which is a low-cost longitudinal imaging method. We have successfully developed orthotopic mouse models employing primary human cancer cells genetically engineered to express Firefly-luciferase (Fluc), providing an ease-of-use, low cost and high-throughput imaging mechanism to monitor tumor growth^13^,^14^.

The aim of the present study was to develop CAM bioluminescent tumor models employing low passage cell cultures obtained from primary PDACs, transduced with lentivirus expressing Fluc. Finally, for a pilot pharmacological study, we treated the CAM tumors with gemcitabine, a standard chemotherapeutic agent used for the treatment of PDAC patients, and with the targeted agent crizotinib, that has been previously shown to interact synergistically with gemcitabine^13^.

## Results

### Establishment of PDAC CAM from primary cultures

**Figure 1:**
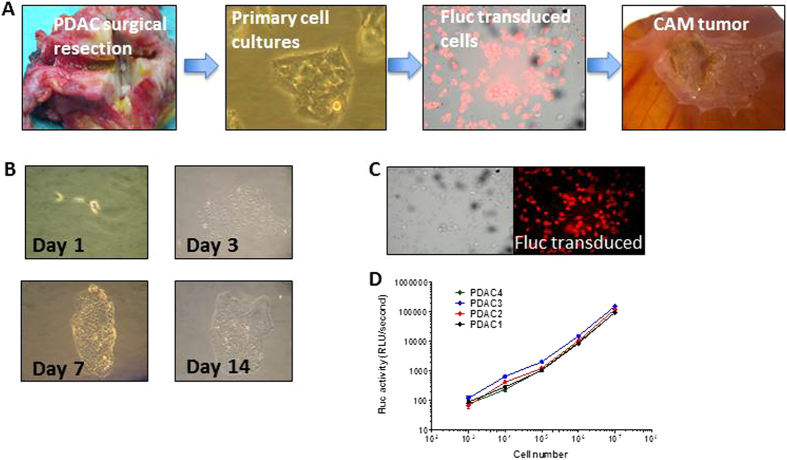
PDAC primary cell cultures were successfully transduced with Fluc and subsequently implanted on the Chick Embryo Chorioallantoic Membrane (CAM) (**A**). Summarizing workflow of the experimental procedures (**B**). Establishment of primary cell cultures (**C**). Representative fluorescence microscopy images of PDAC-3 cells transduced with Fluc (**D**). Increase in the BLI signal Fluc correlates directly with the increasing number of cells, for all four primary established cell lines; y axis: relative light units per second (Rlu/s).

We first sought to develop novel CAM imaging models of PDAC from human primary cultures, as the workflow outlined in Fig. 1A depicts.

Using the above-described protocol, within a few days small areas of epithelial cell growth were observed (Fig. 1B). After approximately two weeks we successfully established primary cell cultures for four primary PDAC tumors (PDAC-1/2/3/4) resected from 10 consecutive patients undergoing pancreaticoduodenectomy (40% efficiency). These cells adhered to the tissue culture Petri dishes and tissue culture flasks as monolayers and plating efficiency increased with passage number, while doubling time decreased. Cell cultures were allowed to grow to 70% confluence. All subsequent experiments described in the present study utilized cells collected during passages 5 to 8.

All the primary PDAC cells were successfully transduced with a Fluc expressing lentiviral vector, with transduction efficacy >90% (Fig. 1C). Further control over multiple passages demonstrated the stability of transduction (data not shown). The BLI signal correlated proportionately with the cell number (Fig. 1D).

**Figure 2:**
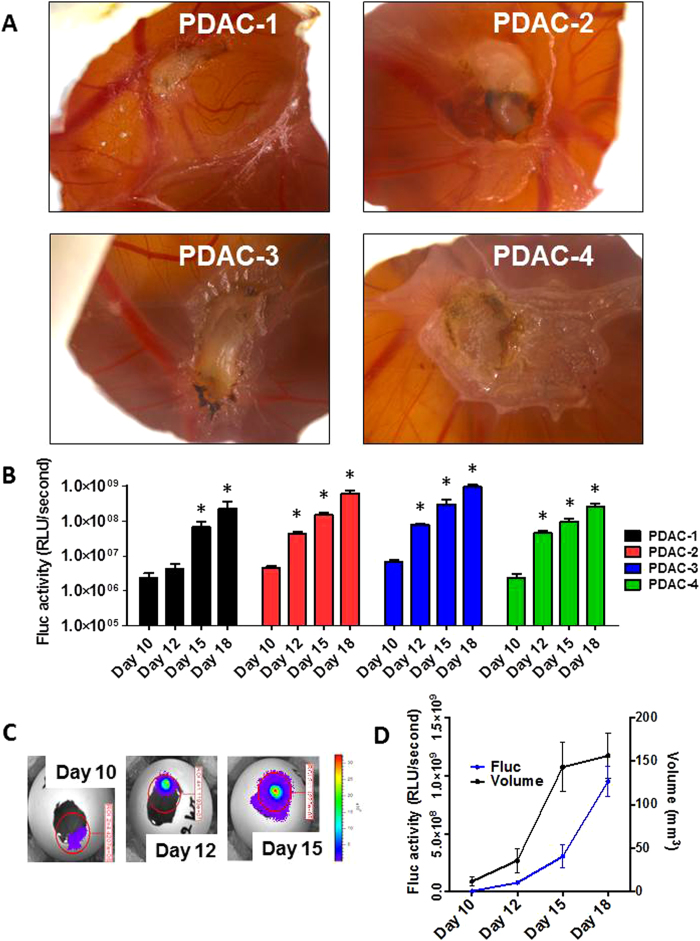
Tumorigenesis on the CAM membrane from primary cells and monitoring of tumor growth via bioluminescence (**A**). Representative photos from the tumors growing on the CAM from the 4 different primary cell lines (**B**). Detection of BLI, indicative of viable cells and subsequent correlation of tumor cell proliferation, indicative of tumor growth, with time (**C**). Representative images of charge-coupled device (CCD) camera images of eggs bearing Fluc-mCherry PDAC-3 cells; days: days after implantation. (**D**) Fluc signals of the CAM in the PDAC-3 model correlated with the volumes detected with caliper (Spearman R^2^ = 0.83); day: days after implantation; y axis: relative light units per second (Rlu/s). Error bars, SEM. *p < 0.05 vs. Day 10.

The optimization process was initiated with 5 to 10 × 10^6^ cells inoculated on the CAM but ultimately, comparable tumor growth was established when downscaling to 1 to 3 × 10^6^ cells, as previously reported for ascites-derived primary PDAC cells^15^. All four primary transduced PDAC cell cultures successfully engrafted, tumors formed and grew over time. These tumors predominantly developed as plaques below the CAM surface, initially avascular until neovascularization occurred by penetration of the pre-existing CAM vasculature into the tumor tissue. Punctiform capillary bleeding was observed through all the plaques. The different PDAC primary cultures showed a distinct phenotypical growth pattern on the CAM. In particular, PDAC-1, PDAC-2, and PDAC-4 formed spreading plaques, with a distinct increase of their surface from EDD8 while the tumors formed from PDAC-3 cells tended to contract the chorionic epithelium (Fig. 2A). At the end of the experiment, tumor-grafting rate exceeded 80% and tumors reached a volume of approximately 150 mm^3^.

### Monitoring of CAM tumors growth via bioluminescence

In all the PDAC tumors growing on the CAM, the BLI was detected for more than one week, indicating viable cells. Importantly, in most tumors the signal grew significantly over time (Fig. 2B), as shown by the increase in Fluc signal intensities in a representative CAM injected with the PDAC-3 cells (Fig. 2C). Moreover, the BLI signals were increasing proportionally to the increase in tumor growth as monitored via caliper measurements. Of note, at each time point, the variability of the values detected with the Fluc measurement was lower than that observed for the caliper measurement, as detailed by the PDAC-3 model in the Fig. 2D. Despite the limited number of models (N = 4), these data suggest that Fluc measurement had a higher accuracy, resulting in the detection of a smaller and tighter data spread in PDAC CAM models.

### Histopathological and IHC findings

**Figure 3:**
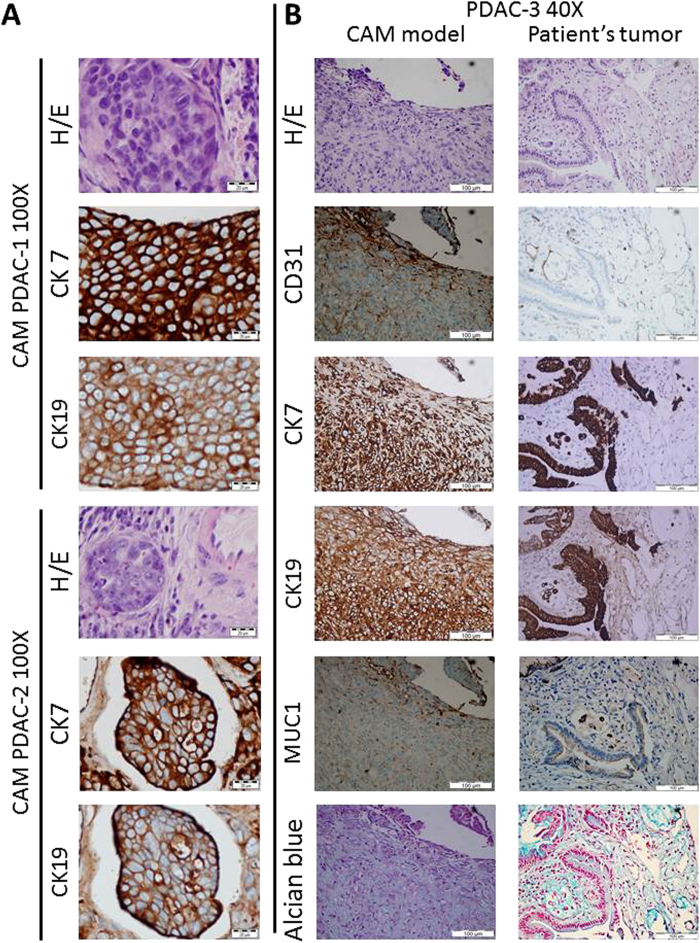
Tumors harvested from the CAM exhibit comparable to the original tumor immunohistochemical stainings (**A**). Representative H/E and cytokeratin CK7 and CK19 stainings of tumor harvested from the CAM models PDAC-1 and PDAC-2 (of note, the 100X magnification clearly shows the counterstaining of the nuclei) (**B**). Representative images from comparative IHC staining for CD31, cytokeratins CK7 and CK19, MUC1 and Alcian Blue in the PDAC-3 tissue specimens, and in the corresponding CAM model.

The H&E staining of resected tumors revealed an organized structure of tumor cells nests within stromal tissue. These tumors also showed the presence of red blood cells, indicative of tumor neovascularization. PDAC transplanted cells became indeed vascularized, and this observational finding was further confirmed by CD31 staining. Subsequently we performed comparative IHC staining in the original human samples demonstrating positive staining for the PDAC markers cytokeratins CK7 and CK19, mucin-1 (MUC1) and Alcian blue (Fig. 3). Similar staining results were observed in the original human tissues. Importantly the percentages of positive/negative cells were highly consistent within individual models.

### Mutation analysis

To investigate the mutational profiles of PDAC-related genes in our CAM tumor models and in the original human tumor, we extracted DNA from laser-microdissected frozen samples, and subjected them to sequencing on selected amplicons for representative tumor-related genes. In particular, the genetic landscape of PDAC is notable for four frequently mutated genes, classifiable as “driver” genes, including *K-RAS, TP53, CDKN2A*/*p16INK4a*, and *SMAD4*/*DPC4*. These four genes are well recognized as contributing to the carcinogenesis and maintenance of PDAC, and the simultaneous determination of their status provides important information regarding disease progression and survival^16^. DNA extracted from frozen samples was successfully amplified in 100% of all the original human tumors and CAM tumors specimens, for each studied exon. The results of these genetic analyses are reported in the Table 1. Activating mutations of *K-RAS* as well as inactivating mutations of *TP53* were found in all the four original human tumors and in their respective CAM tumor models. *CDKN2A*/*p16INK4a* aberrations were detected in all tumors/models, except original tumor-3 and CAM model-3 (i.e., PDAC-3 tumor and CAM model). However, the sequence analysis did not reveal any abnormality in the coding sequences of *SMAD4*/*DPC4* gene in PDAC-1 and PDAC-3 tumors and CAM models. As an adjunct to sequencing, paraffin-embedded samples of the original tumors and matched CAM models were immunolabeled for Cdkn2A, p53 and Smad4 proteins and these results are also reported in Table 1.

### Gene expression of SOX9 and HNF6

A small set of ductal transcription factors, including SRY-related HMG box factor 9 (Sox9) and hepatocyte nuclear factor 6 (Hnf6, also known as Onecut1), have been identified in pancreatic progenitor cells, and a recent study showed their critical role for repression of acinar genes, modulation of acinar-to-ductal metaplasia-associated changes in cell polarity and for activation of ductal genes^17^. Thus, we evaluated the mRNA expression of these genes using RNA extracted from laser-microdissected frozen samples from all the original tumors and CAM models, as described above. *HNF6* mRNA was not detectable in any of these samples, in agreement with a recent study showing that decreased expression of HNF6 is strongly correlated with increased severity of PanIN lesions in samples of human pancreata and is absent from >90% of PDAC^18^.

**Figure 4:**
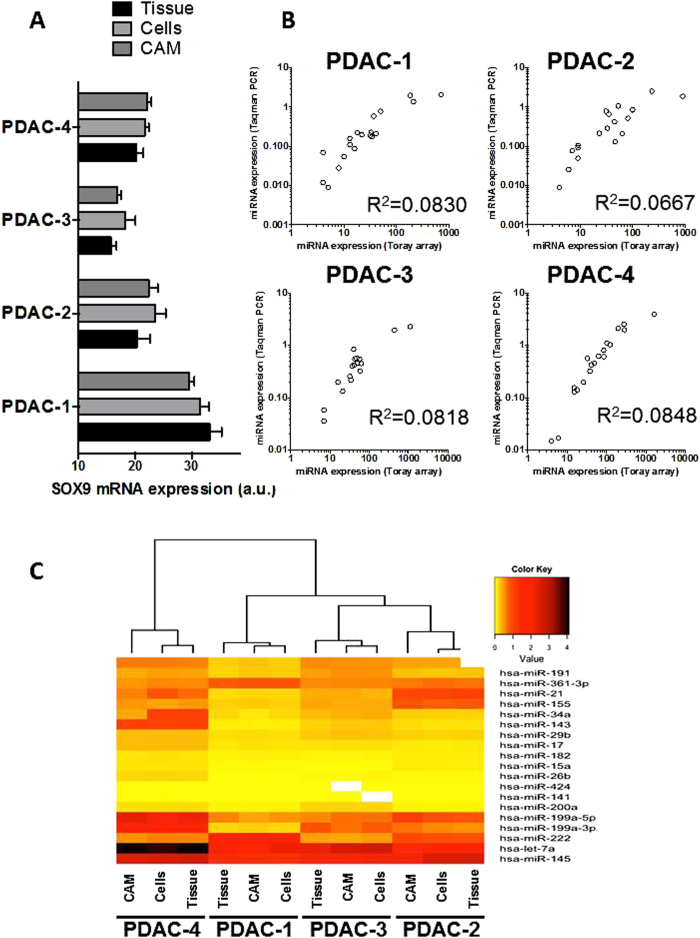
Profiling of *SOX9* and key miRNAs show similar results in PDAC tissues, primary cells and CAM models (**A**). Gene expression levels of *SOX9* in PDAC original tissues, primary cells and CAM models, as detected by quantitative-RT-PCR. (**B)** Comparison of data from miRNA arrays to results of Taqman RT-PCR in all the originating tumor tissues used to establish the primary cultures and the CAM models (**C**). Heat map of the unsupervised hierarchical clustering of miRNA expression levels in the four PDAC models. Error bars, SEM.

Conversely, *SOX9* expression was detectable in all the samples, with values ranging from 15.7 arbitrary unit (a.u.) in the PDAC-3 to 33.2 a.u. in the PDAC-1 tumors (Fig. 4A). The expression in all the tumor samples was significantly higher than that in the normal hTERT-HPNE cells (1.6 a.u.; P < 0.01). Notably, *SOX9* expression values in the four CAM tumor models and in their laser-microdissected original tumors showed a similar pattern and were highly correlated with Spearman analysis (R^2^ = 0.910, P < 0.01).

### MicroRNA profiling

Previous studies suggest that patient-derived xenografts of PDAC retain, to some extent, a gene expression profile similar to the original primary tumors, while this pattern is not detected in conventional cancer cell lines^19^. However, because of the pivotal regulatory role of each miRNA in controlling expression of multiple gene transcripts, expression patterns of 217 miRNAs were found to classify cancer types more accurately than the information based on expression profile of ~16000 mRNAs^20^. Therefore, in the present study we performed a miRNA profiling of our tissues and models.

The signal intensity of all the spots including miRNAs, controls and blanks measured by microarray scanner were detected in duplicate for all the 4 samples assayed by the Toray’s 3D-Gene™ human miRNA chip, and the background subtracted intensities were globally normalized, as described previously^21^.

The following analyses were performed only on 20 miRNAs, selected according to previous studies on their role in PDAC cells and tissues. The statistical analyses to evaluate the comparability to Taqman RT-PCR (Fig. 4B) demonstrated significant correlations between the different datasets, with R^2^ values ranging between 0.667 and 0.848 in the PDAC-2 and PDAC-4 tissues, respectively. Importantly, miRNA expression levels in our samples were comparable to the data that we have reported in a larger cohort of PDAC patients^21^.

PCR was subsequently performed on the corresponding primary cultures and CAM tumors. Unsupervised hierarchical clustering revealed that miRNA expression levels discriminate the four distinct tumor models, where each original tumor tissue clustered together with the ensuing primary culture and the consecutive CAM tumor (Fig. 4C). Moreover, statistical analyses to evaluate the comparability between PCR data in the original tumors and their respective CAM models showed R^2^ values always above 0.85. This indicates that our models shared the same epigenetic characteristics of the human original tumors.

### Gemcitabine and crizotinib inhibited the tumor growth in CAM model

**Figure 5:**
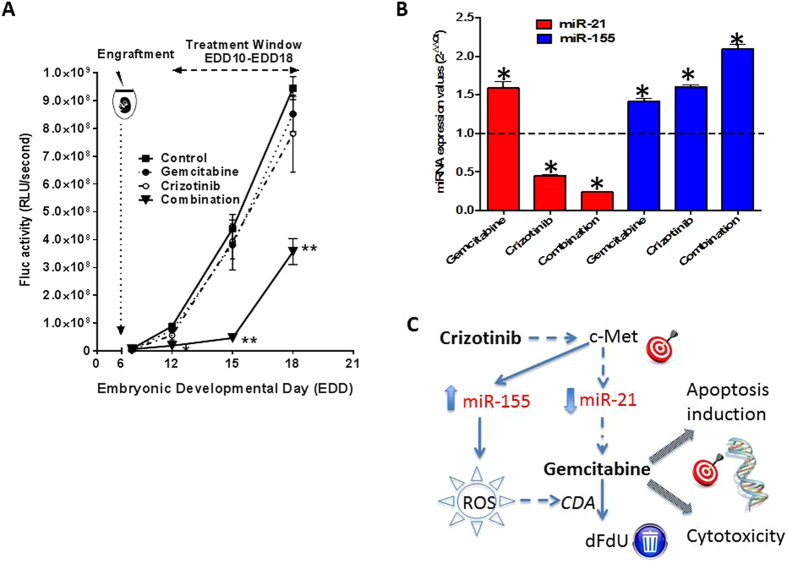
Treatment of tumors growing on the CAM (**A**). Growth curve as detected by BLI, of CAM tumors treated as schematically represented with the designated drugs (**B**). Expression of miR-155 and miR-21 on the CAM tumors treated as indicated and collected on EDD19 (**C**). Schematic hypothesis of the mechanism underlying the synergy among gemcitabine and crizotinib. y axis: relative light units per second (Rlu/s). Error bars, SEM. *p < 0.05, **p < 0.01.

For a pilot pharmacological study, exploring the feasibility of the CAM model for testing antineoplastic agents, we subsequently used the thus far best optimized CAM model, PDAC-3. On EDD10, the eggs were stratified on the basis of BLI intensities into four groups, with comparable mean Fluc activity. They were subsequently treated with vehicle, 100 mg/kg gemcitabine, 25 mg/kg crizotinib, or with the combination of gemcitabine and crizotinib. Treatment of CAM tumors with either gemcitabine or crizotinib monotherapy resulted in modest inhibition of tumor growth as shown by the decrease in mean Fluc intensity, while the inhibition reached statistical significance for the combination treatment (80%, 65% and 63% decrease in tumor growth as denoted by decrease in the BLI intensity on EDD12, 15 and 18 respectively, for the combination treatment versus control; *p* = 0.0136, *p* = 0.0055 and *p* = 0.0051, respectively, Fig. 5A).

Additionally, in the CAM tumors collected on EDD19, we determined how the treatment with gemcitabine and crizotinib affected the expression of miR-155 and miR-21 (Fig. 5B). The expression of miR-21 was upregulated (about 1.5-fold) after gemcitabine, but significantly downregulated (0.5- and 0.2-fold) after exposure to crizotinib and to the gemcitabine-crizotinib combination, respectively. Conversely, the expression of miR-155 was significantly increased after the exposure to both gemcitabine and crizotinib monotherapies, and was additionally upregulated (up to 2.0-fold) by the combination.

## Discussion

In the present article we describe for the first time the establishment of a CAM tumor model from primary PDAC cells, with high tumor engraftment (>80%), that reliably reproduces the growth and histology of PDAC, as well as the expression levels of key miRNAs. Besides that, we provide a step-by-step demonstration of the successful application of this *in ovo* system for testing anticancer drugs.

Despite the advances in molecular biology, in technological applications for genetically engineered mouse models (GEMMs), and the intense focus on the identification of prognostic and predictive biomarkers in “avatar” mice^22^, developments in the area of anticancer drug testing in animal models remain limited. With this study, we propose the repositioning in pancreatic preclinical research of the CAM *in vivo* model, already established for the study of angiogenesis.

A key finding of our study is that all primary PDAC cells, originating from different patients and genetically engineered to express Fluc, were successfully inoculated on the CAM membrane, where tumor growth was reliably monitored by detection of Fluc activity. Importantly, we also addressed one of the debating points in preclinical experiments by pursuing proof that the tumor is the same biological entity as the original tumor, as assessed by comparative histopathological and (epi)genetic analyses.

Necessary prerequisite for advancements in clinical research is the integration in the preclinical setting of validated *in vivo* models that could assist in the investigation of underlying biologic pathways and subsequent implementation of therapeutics. Experimental therapeutic agents have thus far shown limited effects in PDAC trials^1^. One leading hypothesis over the last few years has been that the pronounced stromal microenvironment not only promotes PDAC carcinogenesis but also mediates therapeutic resistance^4^. However, none of the efforts targeting stromal components and pathways have yet led to effective therapies in patients, reducing the impact of tests in GEMMs models. This might be at least partially explained by two recent studies revealing that depletion of stromal cells can prompt a more biologically aggressive form of PDAC with poorly differentiated histology, increased vascularity and proliferation, while depletion of carcinoma-associated fibroblasts induces immunosuppression, associated to epithelial-to-mesenchymal transition^23^,^24^,^25^.

Most recently, Hidalgo and collaborators used “avatar” models as an *in vivo* platform to test treatment strategies suggested by parallel whole-exome sequencing analysis of 25 patients with advanced solid tumors, including seven PDAC patients^22^. However, the use of primary xenografts has still several inherent limitations and deficiencies^26^. The major challenge is the lack of a fully functional human immune system, which could only be reproduced via co-grafting of tumor tissue along with bone marrow stem cells of the same patient, developing extremely complex “humanized mice”^27^. Moreover, continual passaging increases genetic and histopathological differences between patient tumors and xenografts, reducing the heterogeneity reflective of the original neoplasms^28^. Other drawbacks include the special and very high cost maintenance conditions and amount of human and time resources associated with the use of animals, compared to traditional cell line-based systems, as well as the most recent regulations prompting a reduction in the number of animals in many testing establishments worldwide^29^, which hamper the widespread use of these cancer models in drug development. Last but not least, the engraftment of human PDAC cells in mice is a technically demanding procedure, especially for the establishment of orthotopic models, requiring at least one month. This impedes the feasibility of applying these models to guide personalized treatment choices in a timely manner or in a large scale.

In an attempt to address some of these fundamental flaws, new methods using stem-cell based organoid models, tissue engineering or sophisticated 3D cell culture models have been established^30^,^31^. However, these methods are also quite complex and expensive and cannot replace systemic toxicity tests in living organisms. With this study, we show that the CAM model is able to efficiently support the growth of tumor cells, thereby offering an easy and quick model to study primary tumor formation. Similar to murine models, these CAM models can recapitulate all the steps of tumor growth, but in a shorter period of time, since the tumors are detectable after only four days from cancer cell inoculation. Since the lymphoid system is not fully developed until late stages of incubation, the chick embryo serves as a naturally immunodeficient host capable of sustaining grafted tissues and cells without species-specific restrictions, creating a model that bridges the gap between basic *in vitro* systems and more complex animal cancer xenografts.

CAM assay has already been successfully developed into a tumor model for different cancers, including PDAC^32^. However, previously it has been shown that the long-term maintenance in culture of PDAC cell lines, such as BxPC-3, CFPAC-1 and PANC-1 cells^33^,^34^, resulted in distinct and irreversible loss of crucial genetic and biologic properties. This included the occurrence of distinct stem cell populations and complex genomic aberrations, affecting critical signaling pathways^35^,^36^. A more recent study used PDAC primary cell cultures obtained from ascites, demonstrating the establishment of cell masses on the CAM that were consistent with the *in vitro* studies of tumorigenicity of the PDAC primary cultures^37^.

Importantly, microdissection and subsequent sequence analysis revealed that the CAM models harbor the characteristic signature of *K-RAS, TP53, CDKN2A*/*p16INK4a*, and *SMAD4*/*DPC4* status of the original tumors. Furthermore, in the CAM tumors we observed mRNA levels of *SOX9* comparable to the *SOX9* levels detected in the respective original tumors and primary cultures.

Another step we took in the optimization process of this model is the application of BLI, a low-cost longitudinal imaging method. This method has been used in the evaluation of delivered cell response in pancreatic islet stem cell transplantation, as well as in orthotopic models using MiaPaCa-2 cells and primary cultures, where it has been shown that it provides a reliable indicator for quantification of pancreatic tumors^13^,^38^. The currently commonly used application of the ellipsoid formula after caliper measurement for CAM tumor growth estimation, introduces investigator-associated bias and significant error margin, especially for tumors growing underneath the chorioallantoic membrane. The direct analogy of intensity values to the number of tumor cells supports the use of Fluc intensity to estimate tumor cell growth on the CAM and offers a noninvasive accurate method for monitoring tumor growth and progression kinetics.

In a proof-of-principle pharmacological study, we then demonstrated how our novel model could facilitate a faster, *in vivo* screening of therapeutic agents, as well as the analysis of potential epigenetic factors modulating chemosensitivity. Remarkably, this is the first study showing that primary cancer cells engrafted in the CAM could be treated with standard chemotherapy and a targeted agent. For this study we selected one of the models, which was previously characterized by copy-number gain overexpression of c-Met – overactive in approximately 45% of PDAC patients^13^. Combinational treatment with the c-Met/ALK inhibitor crizotinib and gemcitabine significantly reduced PDAC CAM tumor growth. In addition, the analysis of miRNA modulation allowed for the validation of two synergistic mechanisms related to miR-21 downregulation and miR-155 upregulation, as described in the Fig. 5C. MiR-21 expression has been correlated to gemcitabine chemoresistance and reduced apoptosis-induction^39^. Therefore, the inhibition of c-Met by crizotinib, which caused miR-21 downregulation, as reported previously in NSCLC models^40^, might favor gemcitabine cytotoxicity. On the other hand, the upregulation of miR-155 levels might be explained by a feedback mechanism caused by the inhibition of c-Met, which play an important role in tumor-fibroblasts interactions. A recent study suggested indeed that PDAC cells might activate normal adjacent fibroblasts by means of secreted microvesicles containing miR-155^41^. Remarkably, miR-155 upregulation has been associated with increased reactive oxygen species (ROS) levels^42^, which have been implicated in the degradation of cytidine deaminase (CDA) - the main enzyme in gemcitabine catabolism^43^. As a result of the reduced activity of CDA, we and others already reported increased concentration levels and cytotoxic activity of gemcitabine^13^,^43^.

On the basis of these findings, we propose a research pipeline where tumors obtained from biopsies before treatment could be subsequently grown on the CAM in an efficient workflow that requires limited resources and facilities. This approach could deliver in a period spanning of 2 to 3 weeks, a first insight into specific tumor characteristics, enable personalized cancer medicine and testing of drug sensitivity in individual patients and allow for mechanistic insight into the observed effects of tested therapies. In a larger scale, it could also facilitate faster *in vivo* screening processes of anticancer drugs, helping to reduce development time and costs for novel compounds.

In the last decades CAM emerged as an easy-to-use experimental platform for scientists working in bioengineering, morphology, biochemistry, transplant biology, cancer research and drug development^7^. Nonetheless there are also several limitations, such as the reduced number of reagents compatible with avian species, including antibodies and cytokines. Another obvious limitation of this model is the limited time of tumor growth on the CAM. This inarguably precludes from long term follow up and prolonged longitudinal study of treatment effect, while limited information can be collected on the (micro)metastatic potential of the established tumors, which has been described only in some cancer cell types^44^. On the other hand, it has already been shown in animal models that tumor growth in the first 7 days post engraftment predicts overall survival and treatment effect^45^. In addition, this rapidity defers the CAM model from undergoing significant genetic evolutions, so the harvested CAM tumor is not significantly divergent from the original tumor. Another important limitation is that topical application of the designated compound, despite the detectability of the compound intratumorally and in the chicken circulation, does not reflect systemic drug turnover and modification. However, since the CAM is an isolated system, the half-life of several molecules such as small peptides tends to be much longer in comparison to mammalian models, allowing study of compounds available in small quantities.

In conclusion, bioluminescent CAM model of primary PDAC cultures, as discussed and validated in this study, represent a promising preclinical platform, that could directly counsel individualized clinical decisions, bridging the gap among monolayer cell cultures and sophisticated animal models.

## Materials and Methods

### Isolation, culturing and transduction of primary PDAC cells

Four primary PDAC cell lines (PDAC-1, PDAC-2, PDAC-3 and PDAC-4) were isolated from primary PDACs of patients undergoing pancreatoduodenectomy. All the patients provided written informed consent prior to sample collection and the study protocol was approved by the local institutional Ethics Committee at University Hospital of Pisa, Italy (Comitato di Bioetica, Azienda Ospedaliero-Universitaria Pisana, protocol number: 3909, entitled “Farmacogenetica ed epigenetica di determinanti chemioterapici nei tumori pancreatici e correlazione con l’outcome clinico”). Collection of samples was performed in accordance with the relevant guidelines and regulations. Following tissue acquisition, we utilized a modified protocol based on a previously validated method^46^. Non necrotic areas of the excised tumors were minced into 1-mm^3^ cubes and subsequently rinsed in a solution (1 mg/ml) of type XI Collagenase (Sigma-Aldrich, Zwijndrecht, The Netherlands) in primary tissue culture plates (PRIMARIA^TM^ Tissue Culture Flask, Becton Dickinson, NJ) and RPMI-1640 medium (Lonza, Verviers, Belgium), supplemented with 10% heat-inactivated fetal bovine serum (FBS) and 1% streptomycin/penicillin (Gibco, Gaithersburg, MD), and maintained at 37 °C in a humidified incubator. Medium was first removed within 20 minutes after seeding the cells, to eliminate fibroblast contamination. After 18 hours, the cells were harvested and medium was replaced every 3 days, until cell colonies were identified. Trypsinization and medium change removed persistent fibroblasts. After less than 10 passages, the resulting populations of primary PDAC cell cultures were transduced with a lentiviral vector encoding Fluc plus mCherry (Fluc-mCherry), as described previously^14^,^47^. Transduction efficiency of the cells was evaluated using Leica-MM-AF-NX fluorescence microscope (Leica, Wetzlar, Germany) as well as by FACS analysis (Becton Dickinson, San Jose, CA).

### CAM tumor grafts

Fertilized chicken White Leghorn eggs were incubated in a fan-assisted hatching incubator at a temperature of 38 °C and constant air humidity of 70%. Dutch legislation does not necessitate the acquisition of approval from institutional or licensing committee for experiments on the CAM terminated before hatching of the chicken embryo. On Embryonic Developmental Day 6 (EDD6), CAM surface was gently scratched and a total of 1 to 10 × 10^6^ PDAC cells from 4 different primary cultures in suspension with 50% growth factor reduced Matrigel (Becton Dickinson, Breda, The Netherlands) to a total volume of 50 μL were grafted on CAM (N = 10 eggs for each cell line). The eggs were incubated under standard conditions. Tumor volume was followed every 2 or 3 days, and calculated using an external caliper, by the modified ellipsoid formula ½ × (length × width^2^). On EDD19 tumors were collected and paraffin-embedded for the following studies on histopathological characteristics and miRNA levels^7^.

### Monitoring of CAM tumors growth via bioluminescence

Tumor growth was also monitored through BLI, every 2 or 3 days, using Fluc, which catalyzes the oxidative decarboxylation of luciferin in the presence of ATP, O_2_, and Mg^2+^, producing yellow-green light. D–Luciferin (150 mg/kg egg weight) was applied and the BLI signal was evaluated with a charge-coupled device (CCD) camera, equipped with the Xenogen IVIS Lumina System (Xenogen Corp., Alameda, CA). Acquired images were analysed using Living Image 3 software (Xenogen Corp.). Regions of interest were defined using an automatic intensity contour procedure to identify bioluminescence signals with intensities significantly greater than the background. The mean (p/s/cm^2^), standard deviation, and sum of the photon counts in these regions were calculated as a total measurement of Fluc activity. For visualization purposes, bioluminescence images were fused with the corresponding white light image of the egg taken in the chamber with dim polychromatic illumination, using a transparent pseudocolor overlay.

### Histopathological and immunohistochemical analyses

The excised CAM tumors were fixed in 4% paraformaldehyde overnight at 4 °C, and then placed into embedding cassettes and transferred into a tissue embedding station with an increasing graded alcohol series (50%, 70%, 80%, 95% ethanol, xylol and paraffin). Sections of the paraffin embedded tissues (3 μm) were deparaffinized by a decreasing graded alcohol series to double-distilled water (xylol, 95%, 80%, 70%, 50% ethanol, double-distilled water) and then used for histopathological analyses with hematoxylin and eosin (H&E). Immunohistochemistry (IHC) was performed according to standard procedures with a panel of antibodies (cytokeratin 7 (CK7, clone OV-TL 12/30, Dako, Heverlee, Belgium), CK19 (clone EP72, Epitomics, Berkshire, UK), and mucin-1 (MUC1, clone E29, Dako)) routinely used in PDAC diagnosis. Visualization was obtained by BenchMark Special Stain Automation system (Ventana Medical Systems, NY, USA). Furthermore, we performed Alcian Blue staining (Artisan kit, Dako), since this is a marker of high acidic mucin accumulation.

### Mutation analysis

The genetic analysis of *K-Ras, TP53, p16INK4a*/*CDKN2A* and *SMAD4* was performed by Sanger sequencing using DNA extracted from both the original human tumors and the CAM tumors.

Tumor tissues were dissected macroscopically if the neoplastic cellularity was at least 60%, or microscopically using the LMD7000 instrument (Leica), for cases with low neoplastic cellularity, as described previously^47^. Genomic DNA was extracted from each sample using Trizol, following manufacturer instructions (Life Technologies, Breda, The Netherlands) or QIAmp DNA Micro Kits if microdissected (Qiagen, Hilden, Germany). We used 20 ng of genomic DNA, as template in nested PCR reactions to amplify DNA fragments corresponding to exons 1 and 2 of *K-RAS, TP53* exons 5–9, *CDKN2A*/*p16INK4a* exons 1 and 2, and *DPC4*/*SMAD4* exons 0–11. The PCR protocol and the sets of primers have been described in detail previously^16^,^48^. PCR products were purified using a presequencing kit (Amersham Biosciences, Roosendaal, The Netherlands) and sequenced with both forward and reverse primers using the BigDyeTM Terminator v3.1 Cycle Sequencing Kit (Applied Biosystems, Foster City, CA), with the ABI PRISMTM 3100 Genetic analyzer (Applied Biosystems). Mutation analysis, confirmation and determination of somatic status were carried out by sequencing independent PCR products using matched normal tissues from the same patient, DNA derived from the primary cultures and DNA from PDAC tumor cells with well-known genetic profiles.

Additional IHC studies were performed using antibodies to Cdkn2A (clone E6H4, MTM Laboratories, Heidelberg, Germany), p53 (Bp-53-11, Ventana) and Smad4 (clone B8, Santa Cruz Biotechnology, Santa Cruz, CA) proteins, as reported previously^16^. Nuclear labeling of Cdkn2A was scored as intact (positive, indicating the presence of an intact gene) or lost (negative, indicating a deletion, inactivating mutation or promoter hypermethylation); p53 immunolabeling was considered abnormal when it showed nuclear accumulation of this protein in ≥30% of the neoplastic cells compared to adjacent normal cells, or if the neoplastic cells showed a virtual absence of immunolabeling compared to immediately adjacent normal cells suggesting the presence of an intragenic deletion, nonsense or frameshift mutation; nuclear labeling of Smad4 was scored as intact (positive, indicating the presence of an intact gene) or lost (negative, indicating a deletion or inactivating mutation of the gene has occurred). Normal islets for Cdkn2A and normal acinar cells, islets, lymphocytes and stromal cells for p53 and Smad4 were regarded as internal positive controls for each case.

### Quantitative RT-PCR

In order to evaluate the key ductal transcription factors Sox9 and Hnf6, we performed quantitative RT-PCR, using Taqman^®^ technology and the ABI PRISM 7500 Sequence Detection System instrument equipped with the SDS version 2.3 software (Applied Biosystems). Total RNAs were extracted from laser-microdissected frozen samples, using the QIAamp RNA Micro Kit (Qiagen). The quantity and purity of extracted DNA were assessed at 260-280 nm with the NanoDrop^®^-1000-Detector (NanoDrop-Technologies, Wilmington, NC), and cDNA was obtained with the DyNAmo cDNA Synthesis Kit (Thermo Scientific, Vantaa, Finland), according to the manufacturers’ instructions. Forward and reverse primers and probes were designed and produced by Applied Biosystems for *SOX9* (Hs00165814_m1) and *HNF6* (Hs00413554_m1). PCR was carried out in a 25 μl-reaction volume that contained 25 ng of cDNA, 1× TaqMan Universal PCR Master Mix, and the primer and probe sets. We performed a preliminary analysis of three housekeeping genes (*β-actin, GAPDH* and *Beta-2-microglobulin*) in primary PDAC cultures. Since the values of *β-actin* were the closest to the geometric mean values of these housekeeping genes, we used this housekeeping for the normalization of all following analyses. Preliminary experiments were also carried out with dilutions of cDNA obtained from Quantitative PCR Human Reference Total RNA (Stratagene, La Jolla, CA) to demonstrate that the efficiencies of amplification of the target and reference genes are approximately equal and to determine the absolute value of the slope of standard cDNA concentration versus CT, where CT is the threshold cycle, as reported previously^13^.

### MicroRNA (miRNA) profiling

We utilized Toray’s 3D-Gene^®^ miRNA oligo chip v.16 (Toray Industries, Japan) to analyze human miRNA expression profiling of the FFPE sections of the original tumors, as described previously^21^. Briefly, 500 ng total RNA extracted from the PDAC tissues was analyzed for miRNA profiling using microarray, 3D-Gene^®^ miRNA oligo chip v.16 (Toray Industries) according to the manufacturer’s protocol vE1.10. Microarray was scanned and the obtained images were numerated using 3D-Gene^®^ scanner 3000 (Toray Industries). The expression level of each miRNA was globally normalized using the background-subtracted signal intensity of the entire miRNAs in each microarray.

The reproducibility of these results was tested by Taqman RT-PCR, selecting 18 miRNAs (hsa-let-7a, hsa-miR-141, hsa-miR-143, hsa-miR-145, hsa-miR-17, hsa-miR-182, hsa-miR-191, hsa-miR-199a-5p, hsa-miR-200a, hsa-miR-200b, hsa-miR-222, hsa-miR-29b, hsa-miR-34a, hsa-miR-424, hsa-miR-15a, hsa-miR-199a-3p, hsa-miR-26b, hsa-miR-361-3p) previously showing at least a three-fold modulation in expression in a wide panel of PDAC cell lines^49^. Furthermore, we included in our analysis two miRNAs that have been commonly associated with PDAC pathogenesis and gemcitabine chemoresistance, namely miR-155 and miR-21^46^,^50^.

These miRNAs were then evaluated in the PDAC cells growing as monolayer and in the PDAC tumors harvested from the CAM. For RNA extraction in both the cells and in the CAM tumors the Ambion-RecoverAll kit was used (Thermo Fischer Scientific, Waltham, MA).

### Treatment of the CAM tumors

Tumors originating from the primary PDAC-3 cells were treated daily from EDD10 until EDD18 with either vehicle, or 100 mg/kg gemcitabine (in sterile water), or 25 mg/kg crizotinib (dissolved in DMSO and 0.5% methylcellulose), or with the combination of gemcitabine and crizotinib. The drugs were applied topically on the CAM, in close proximity to the tumor and the total doses for all schedules were calculated based on the weight of the chicken embryo at EDD10.

### Statistical analysis

Statistical tests were performed in all the experiments. In particular, experiments were repeated at least twice and data were expressed as mean values ± standard error of the mean (S.E.M.), and analyzed by Student’s t-test or ANOVA followed by the Tukey’s multiple comparison test. The level of significance was *P* < 0.05.

## Additional Information

**How to cite this article:** Rovithi, M. *et al*. Development of bioluminescent chick chorioallantoic membrane (CAM) models for primary pancreatic cancer cells: a platform for drug testing. *Sci. Rep.* **7**, 44686; doi: 10.1038/srep44686 (2017).

**Publisher's note:** Springer Nature remains neutral with regard to jurisdictional claims in published maps and institutional affiliations.

## Figures and Tables

**Table 1:** Genetics of original human tumors and matched CAM tumors, as detected by sequencing and IHC analyses.

Sample	K-RAS	TP53	p53 status	CDKN2A/p16INK4a	Cdkn2A status	SMAD4/DPC4	Dpc4 status
Original tumor-1	p.Q61H	p.R282G	Mutant	p.R58X	Lost	WT	Intact
CAM tumor-1	p.Q61H	p.R282G	Mutant	p.R58X	Lost	WT	Intact
Original tumor-2	p.G12V	p.V173L	Mutant	HD	Lost	HD	Lost
CAM tumor-2	p.G12V	p.V173L	Mutant	HD	Lost	HD	Lost
Original tumor-3	p.G12D	p.I195T	Mutant	WT	Intact	WT	Intact
CAM tumor-3	p.G12D	p.I195T	Mutant	WT	Intact	WT	Intact
Original tumor-4	p.G12D	p.R273H	Mutant	HD	Lost	HD	Lost
CAM tumor-4	p.G12D	p.R273H	Mutant	HD	Lost	HD	Lost

Abbreviations: HD, homozygous deletion; WT, wild type.
